# MOSES-assisted versus conventional holmium laser enucleation of the prostate for benign prostatic hyperplasia: a pooled study

**DOI:** 10.3389/fmed.2026.1946428

**Published:** 2026-10-14

**Authors:** Puze Wang, Jinze Li, Youlong Huili, Yuchang Tian, Chongzhou Liao, Dong Ren, Huawei Liu, Dong Lv, Lei Xu

**Affiliations:** 1 Department of Urology, People's Hospital of Deyang City, Chengdu University of Traditional Chinese Medicine, Deyang, China; 2 Department of Urology, Deyang Hospital Affiliated Hospital of Chengdu University of Traditional Chinese Medicine, Deyang, China

**Keywords:** benign prostatic hyperplasia, HoLEP, MOSES, efficacy, safety

## Abstract

**Purpose:**

With the widespread adoption of holmium laser enucleation of the prostate (HoLEP), increasing attention has focused on whether pulse-modulation technologies can further improve procedural efficiency and perioperative outcomes. We therefore conducted an updated systematic review and meta-analysis comparing MOSES-assisted HoLEP (MoLEP) with conventional HoLEP for benign prostatic hyperplasia (BPH).

**Methods:**

PubMed, Embase, Web of Science, and the ClinicalTrials.gov were searched through September 2026 for comparative studies evaluating MoLEP versus conventional HoLEP. Study selection and data extraction were performed according to predefined criteria. Pooled analyses were conducted using R (version 4.4.1), with subgroup and sensitivity analyses undertaken where appropriate.

**Results:**

Ten studies comprising 3,177 patients (1,599 MoLEP and 1,578 conventional HoLEP) were included. MoLEP was associated with shorter enucleation time (MD −7.45 min, 95% CI −11.05 to −3.85; *P* < 0.001), hemostasis time (MD −3.79 min, 95% CI −5.93 to −1.65; *P* < 0.001), and operative time (MD −12.09 min, 95% CI −18.01 to −6.18; *P* < 0.001). No significant between-group differences were observed in enucleated weight (MD 1.01 g, 95% CI −3.20–5.21; *P* = 0.639), catheterization time (SMD −0.13, 95% CI −0.35–0.09; *P* = 0.238), or length of stay (SMD −0.23, 95% CI −0.52–0.05; *P* = 0.113). MoLEP was associated with a generally lower incidence of postoperative complications than conventional HoLEP (*P* < 0.001). Regarding functional outcomes, statistically significant between-group differences were observed in PVR at 1 and 6 months, QoL at 3 months, and IPSS at 6 months, whereas other functional outcomes did not differ significantly between groups.

**Conclusions:**

The current evidence suggests that MoLEP may improve perioperative efficiency and reduce postoperative complications, while early functional outcomes remain broadly comparable with conventional HoLEP. Further high-quality studies with longer follow-up are needed to confirm these potential benefits.

**Systematic Review Registration:**

ID: CRD420251177474.

## 1 Introduction

Over the past two decades, anatomic endoscopic enucleation of the prostate (AEEP) has transitioned from a specialist technique to routine practice, with holmium laser enucleation of the prostate (HoLEP) as the most established and extensively studied modality since its first description in 1998 (1, 2). Guideline recommendations reflect this maturity: the European Association of Urology (EAU) endorses AEEP particularly for large prostates (>80 mL), while the American Urological Association (AUA) supports AEEP irrespective of gland size or laser source (3, 4).

Rapid advances in laser physics and pulse delivery have paralleled this clinical evolution. In particular, MOSES technology, a pulse-modulation mode, aims to improve intraluminal energy coupling, stabilize the endoscopic field, and enhance coagulative efficiency without increasing delivered energy (5, 6). Within HoLEP, these properties are hypothesized to yield clearer dissection planes, less bleeding, and more efficient hemostasis, thereby refining the technical conduct of enucleation (7).

An expanding body of clinical research has compared MOSES-assisted HoLEP (MoLEP) with conventional HoLEP. Individual studies have generally suggested improved enucleation and hemostasis efficiency with MOSES technology while showing broadly comparable safety and functional outcomes, although estimates for operative time and other perioperative endpoints remain heterogeneous and may be influenced by prostate size, surgeon experience, enucleation technique, and reporting practices (8–10). Several meta-analyses have subsequently synthesized this early evidence and reached broadly similar conclusions regarding perioperative efficiency, while postoperative functional outcomes were less consistent (11–13).

However, the available evidence base has continued to evolve, with additional comparative cohorts published after these earlier syntheses. Moreover, potential overlap among institutional patient populations was not consistently addressed, and postoperative functional outcomes were often pooled across different follow-up intervals. These limitations may affect the precision and clinical interpretation of previous estimates. Accordingly, we conducted an updated systematic review and meta-analysis comparing MoLEP with conventional HoLEP across perioperative efficiency, safety, and early functional outcomes, while reassessing potentially overlapping cohorts and evaluating functional outcomes at defined postoperative time points.

## 2 Methods

### 2.1 Search strategy

This systematic review and meta-analysis followed the 2020 Preferred Reporting Items for Systematic Reviews and Meta-Analyses (PRISMA) and Assessing the methodological quality of systematic reviews 2 (AMSTAR 2) guidelines (14, 15). We systematically searched PubMed, Embase, Web of Science, and ClinicalTrials.gov for English-language clinical studies reporting outcomes of MoLEP for BPH in adults, from database inception to September 2026. Search terms combined Medical Subject Headings (MeSH) and free-text keywords related to BPH and MoLEP. Reference lists of all eligible studies and relevant reviews were manually screened to identify additional records. Two reviewers independently screened titles/abstracts and full texts after deduplication, with discrepancies resolved by consensus and adjudication by a third reviewer when required. The protocol was prospectively registered in the International Prospective Register of Systematic Reviews (PROSPERO) database (Registration ID: CRD420251177474).

### 2.2 Selection criteria

Studies were eligible for inclusion if they met the following criteria: (P) adult men with benign prostatic hyperplasia (BPH); (I) treatment with MoLEP; (C) a comparator group undergoing conventional HoLEP, including either a concurrent or historical control cohort; (O) reporting at least one perioperative, safety, or functional outcome; and (S) Cs (RCTs) or prospective or retrospective comparative studies.

Exclusion criteria were case reports, letters, editorials, conference abstracts, reviews, animal studies, genuinely non-comparative designs without an eligible conventional HoLEP comparator, pediatric populations, duplicate publications, studies lacking relevant outcome data, and studies in which the enucleation technology was not clearly specified or MoLEP and conventional HoLEP data could not be disaggregated.

When multiple publications originated from the same institution and had overlapping or potentially overlapping recruitment periods or patient populations, only one report was retained for quantitative synthesis to avoid double-counting. Priority was given to the report providing the most comprehensive clinical outcome data and most directly addressing the comparison between MoLEP and conventional HoLEP.

### 2.3 Data extraction

Data extraction was performed independently by two reviewers using a piloted form. We extracted study-level information, including first author, publication year, country, study design, recruitment period, procedure, enucleation technique, fibre size, number of patients, and follow-up duration; baseline characteristics, including age and baseline prostate volume; perioperative variables, including operative time, enucleation time, hemostasis time, enucleated or resected tissue weight, catheterization time, and length of stay; complications according to the Clavien-Dindo classification (16); and functional outcomes, including International Prostate Symptom Score (IPSS), quality of life (QoL), maximum urinary flow rate (Qmax), and postvoid residual volume (PVR). Functional outcomes were assessed at 1, 3, and 6 months where available. For multi-arm studies including more than one eligible MOSES intervention arm and a single conventional HoLEP comparator, the eligible MOSES arms were combined before quantitative synthesis to avoid double-counting of the shared control group. For continuous outcomes, group means and standard deviations were combined using standard formulas, whereas event counts and sample sizes were summed for dichotomous outcomes. Study recruitment periods, institutional affiliations, and author groups were cross-checked to identify overlapping or potentially overlapping patient populations before quantitative pooling. Discrepancies in data extraction were resolved by consensus, with a third reviewer adjudicating when necessary.

### 2.4 Statistical analysis

Statistical analyses were performed using R software (version 4.4.1). Continuous outcomes were pooled as mean differences (MDs) or standardized mean differences (SMDs), as appropriate, with 95% confidence intervals (CIs), whereas dichotomous outcomes were pooled as odds ratios (ORs) with 95% CIs. When continuous outcomes were reported as medians with ranges or interquartile ranges (IQRs), the sample mean was estimated using the method proposed by Luo et al. (17), and the SD was estimated using the method proposed by Wan et al. (18), according to the available sample size and summary statistics (median, quartiles, and/or range). Original mean ± SD data were preferentially used whenever available, and conversion was performed only when necessary for quantitative pooling. Continuous variables measured on the same scale were synthesized using MDs. Because perioperative outcomes, including length of stay (LOS) and catheterization time, were reported using different units and individual-level data were unavailable, these outcomes were synthesized using SMDs. Dichotomous variables were pooled as ORs with 95% CIs.

Heterogeneity was assessed using Cochran's *Q* test and the I^2^ statistic. A random-effects inverse-variance model with REML estimation was used when I^2^ ≥ 50% or *p* < 0.10 for the Q test; otherwise, a fixed-effect model was applied. Sensitivity analyses, including leave-one-out analyses, were performed when substantial heterogeneity was present. Subgroup analyses were conducted by study design (RCT vs. nonrandomized studies) and enucleation technique (en-bloc vs. lobe-based). Studies with unclassifiable enucleation techniques were excluded from the technique subgroup analysis but retained in the overall analysis. Subgroup differences were assessed using formal interaction tests. To evaluate bias from converting median-based data to mean ± SD, sensitivity analyses excluding studies requiring conversion were performed. Potential small-study effects were explored using funnel plots and Egger's regression. Because fewer than 10 studies were available for each outcome, these analyses were considered exploratory and interpreted cautiously. A two-sided *p* < 0.05 was considered statistically significant.

### 2.5 Risk of bias assessment

In accordance with the Cochrane Handbook for Systematic Reviews of Interventions, we assessed methodological quality using the Risk of Bias 2 (RoB 2) tool for RCTs and the Risk of Bias in Nonrandomized Studies of Interventions (ROBINS-I) tool for nonrandomized comparative studies (19, 20). Two reviewers appraised risk of bias independently, with disagreements resolved by discussion and, when necessary, adjudication by a third reviewer.

## 3 Results

### 3.1 Study characteristics

The database search identified 260 records, of which 127 remained after duplicate removal. After screening and full-text eligibility assessment, 10 comparative studies (8–10, 21–27) published between 2021 and 2026 were included in both the qualitative synthesis and quantitative meta-analysis, as shown in the PRISMA flow diagram (Figure 1). The included studies comprised two randomized controlled trials, seven retrospective comparative studies, and one prospective cohort study using a retrospective historical control. A total of 3,177 participants were included, with 1,599 undergoing MoLEP and 1,578 undergoing conventional HoLEP. Enucleation techniques included en-bloc, lobe-based, and two-lobe approaches, and a 550-*μ*m laser fibre was used in most studies reporting fibre size. Among studies reporting postoperative follow-up, most evaluated outcomes within 6 months, whereas one study reported follow-up to 12 months. The detailed characteristics of the included studies are summarized in Table 1.

**Figure 1:**
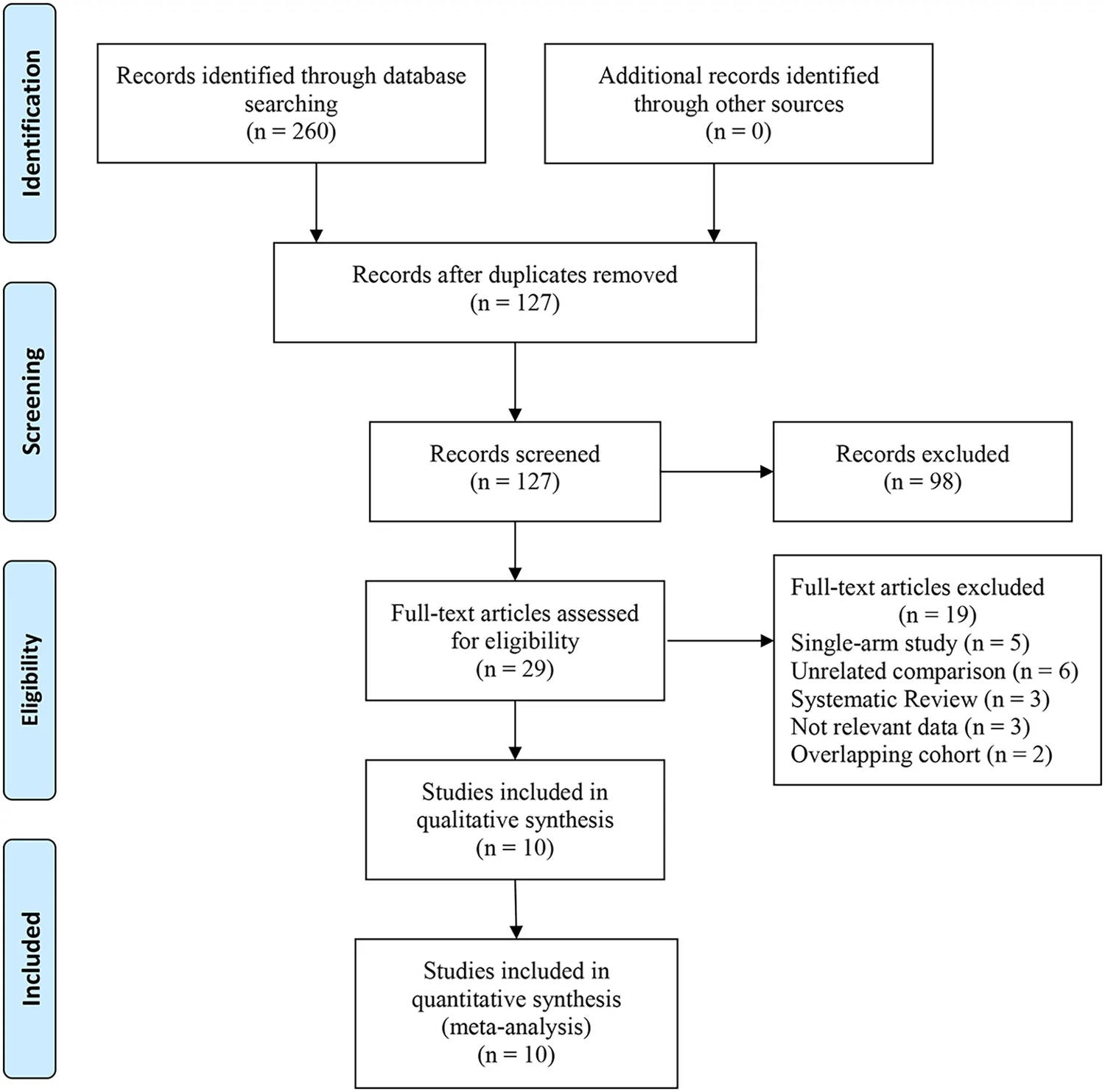
PRISMA flowchart of studies included in the systematic review.

**Table 1:** Characteristics of studies comparing MoLEP versus HoLEP.

Study	Country	Study design	Procedure	Recruitment period	Enucleation technique	Fibre size (μm)	Patients (n)	Age (years)	Prostate size	Follow-up
Kavoussi, et al. 2021 (8)	USA	RCT	MoLEP HoLEP	2020.01–2020.12	Two-lobe	550/550	30 30	70.1 ± 8.2 68.6 ± 6.1	131 ± 41 153 ± 58	6 W
Klett, et al. 2021 (18)	USA	RS	MoLEP HoLEP	2018.08–2021.02	En-bloc	550/550	255 180	71 (66–75) 72 (67–76)	98 (69–124) 89 (65–120)	NA
Nottingham, et al. 2021 (9)	USA	RS	MoLEP HoLEP	2018.08–2019.01	Lobe	550/550	54 50	72 ± 8 71 ± 9	NA	3 M
Noureldin, et al. 2023 (10)	Canada	RS	MoLEP HoLEP	HoLEP: 2018.08–2019.01; MoLEP: from 2019.06^b^	Lobe	550/550	62 28	71.4 ± 7 71.5 ± 7	109.5 ± 30.8 115.6 ± 38.5	6 M
Socarrás, et al. 2023 (20)	Spain	PS/HC	MoLEP HoLEP	2019.04–2019.11^c^	En-bloc	550/550	80 137	66.7 ± 13.2 66.3 ± 8	86.7 ± 50 75.8 ± 42.3	12 M
Tong, et al. 2024 (21)	China	RCT	MoLEP HoLEP	2022.03–2022.12	Lobe	550/550	38 42	69 ± 8.3 68.6 ± 7.8	92.2 ± 19.6 86.7 ± 18.5	6 M
Hartung, et al. 2024 (22)	Germany	RS	MoLEP HoLEP	2021.01–2023.03	En-bloc	550/550	117 117	70 ± 9 71 ± 8	104 ± 59 102 ± 53	NA
Veveloyiannis, et al. 2025 (23)	Greece	RS	MoLEP HoLEP	2022.05–2025.03	En-bloc	550/550	680 688	70 (67–74) 71 (64–77)	127 (103–160) 123 (97–152)	6 M
Lacroix, et al. 2026	France	RS	MoLEP HoLEP	MoLEP: 2023.06–2025.04; HoLEP: 2015–2025	NA	NA	100 100	71 (66.5–76) 72 (67.5–75.5)	79.8 (60–100) 79.8 (59–105)	3 M
Velasquez-Ospina, et al. 2026^a^	USA	RS	MoLEP HoLEP	2022.06–2024.05	En-bloc	550/550	183 206	71.6 ± 7.4 74.2 ± 8.2	131.4 ± 73.8 124.2 ± 68.3	6 M

HoLEP, holmium laser enucleation of the prostate; MoLEP, MOSES HoLEP; RS, retrospective study; RCT, prospective randomised study; PS/HC, prospective single-arm cohort with retrospective historical control; M, month; W, week; NA, not available.

a For Velasquez-Ospina et al. (2026), the MOSES 1.0 (*n* = 126) and MOSES 2.0 (*n* = 57) groups were combined into a single MoLEP group (*n* = 183) for the present meta-analysis to avoid double-counting of the shared standard HoLEP comparator (*n* = 206).

b For Nottingham et al., the standard HoLEP cohort underwent surgery between August 2018 and January 2019, whereas recruitment of the M2-HoLEP cohort began in June 2019; the end date of M2-HoLEP recruitment was not explicitly reported.

c For Socarrás et al., the recruitment period was obtained from the preceding conference report describing the same 217-patient cohort.

### 3.2 Risk of bias assessment

The risk-of-bias assessment is summarized in Supplementary Figure S1. Among the two randomized controlled trials assessed using RoB 2, Kavoussi et al. (8) was judged to be at low risk of bias, whereas Tong et al. (23) was judged as having some concerns, primarily because 20 of the 100 randomized participants were not included in the final analysis. Among the eight nonrandomized comparative studies assessed using ROBINS-I, six were judged to have a moderate risk of bias, mainly because of residual confounding, nonrandomized treatment allocation, or the use of historical comparator cohorts. Veveloyiannis et al. (25) was judged to have a serious risk of bias because of post-treatment exclusions and loss to follow-up that could have influenced selection into the analyzed cohort. Velasquez-Ospina et al. (27) was also judged to have a serious risk of bias, particularly for functional outcomes, because of substantial attrition and available-case analysis at later follow-up. No study was judged to be at critical risk of bias.

### 3.3 Perioperative outcomes

Pooled analysis of seven studies (1,061 MoLEP vs. 1,092 HoLEP) showed a significantly shorter enucleation time with MoLEP (MD −7.45 min, 95% CI −11.05 to −3.85, *p* < 0.001, I^2^ = 75.2%; Figure 2A). Six studies involving 381 MoLEP and 404 HoLEP patients demonstrated a shorter hemostasis time with MoLEP (MD −3.79 min, 95% CI −5.93 to −1.65, *p* < 0.001, I^2^ = 90.9%; Figure 2B). Operative time was also shorter with MoLEP across five studies (961 vs. 1,022 patients; MD −12.09 min, 95% CI −18.01 to −6.18, *p* < 0.001, I^2^ = 71.4%; Figure 2C). In contrast, no significant between-group difference was observed in enucleated prostate weight across nine studies (1,482 vs. 1,461 patients; MD 1.01 g, 95% CI −3.20–5.21, *p* = 0.639, I^2^ = 72.4%; Figure 3A). Similarly, catheterization time did not differ significantly between groups in five studies (945 vs. 1,014 patients; SMD −0.13, 95% CI −0.35–0.09, *p* = 0.238, I^2^ = 66%; Figure 3B), nor did length of stay in six studies (1,128 vs. 1,220 patients; SMD −0.23, 95% CI −0.52–0.05, *p* = 0.113, I^2^ = 85.1%; Figure 3C).

**Figure 2:**
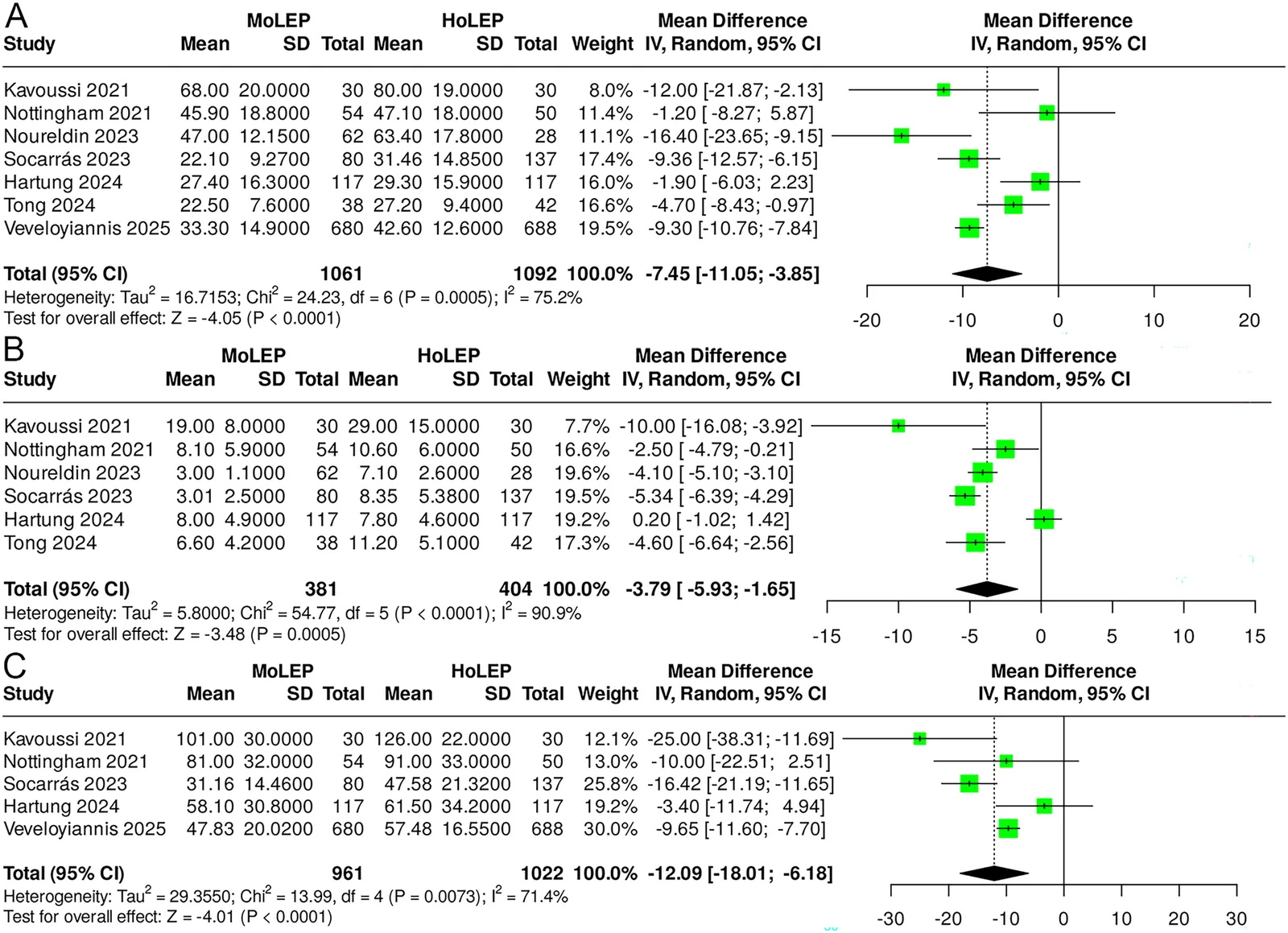
Forest plot representing analysis of perioperative outcomes. **(A)** enucleation time, **(B)** hemostasis time, **(C)** operative time.

**Figure 3:**
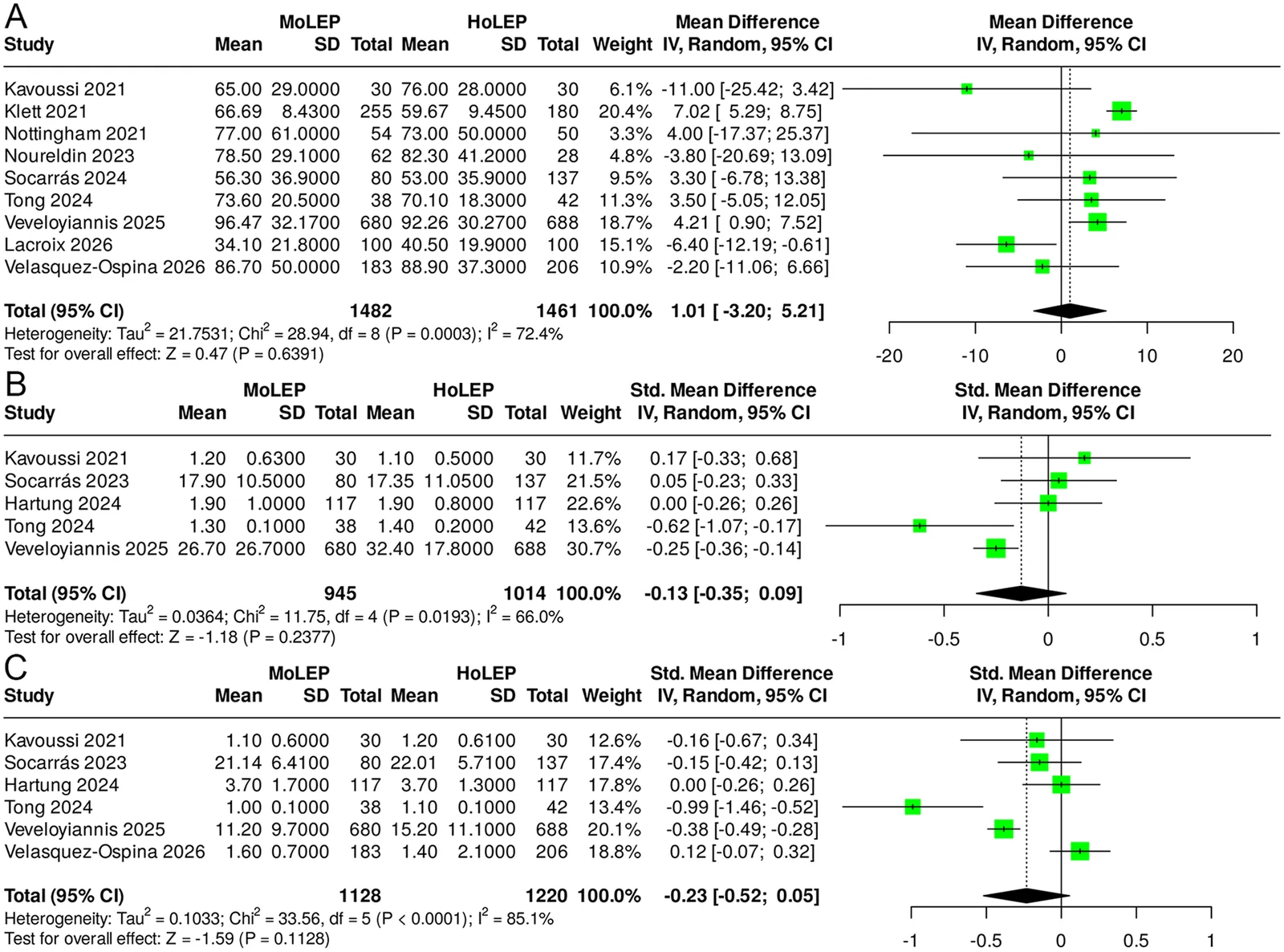
Forest plot representing analysis of perioperative outcomes. **(A)** enucleated weight, **(B)** catheterization time, **(C)** length of stay.

Subgroup analysis by enucleation technique showed no significant differences between en-bloc and lobe-based approaches for enucleation time, hemostasis time, operative time, catheterization time, or length of stay (all *P* for subgroup difference > 0.05; Table 2). A significant interaction was observed for enucleated weight (*P* for subgroup difference = 0.003): MoLEP was associated with greater enucleated weight in the en-bloc subgroup (MD 4.79 g, 95% CI 1.63–7.96), whereas no significant difference was observed in the lobe-based subgroup (MD −1.06 g, 95% CI −8.79–6.67). Subgroup analysis by study design yielded broadly consistent findings between RCTs and nonrandomized comparative studies (Table 3). No significant subgroup differences were observed for enucleation time (*P* = 0.519), hemostasis time (*P* = 0.216), enucleated weight (*P* = 0.590), catheterization time (*P* = 0.748), or length of stay (*P* = 0.275). For operative time, however, the between-subgroup difference reached statistical significance (*P* = 0.047), with a larger reduction in the RCT subgroup (MD −25.00 min, 95% CI −38.31 to −11.69) than in the nonrandomized subgroup (MD −10.47 min, 95% CI −15.81 to −5.13). Because the RCT subgroup for operative time contained only one study, this finding should be interpreted cautiously.

**Table 2:** Results of the primary analysis and subgroup analyses for outcomes.

Outcomes	MD/OR	95% CI	P	Heterogeneity		P* difference
				I² (%)	P	
Enucleation time	**−7**.**45**	**−11.05, −3.85**	**<0**.**001**	**75**	**<0**.**001**	
En-bloc	−7.13	−11.69, −2.56	0.002	82	0.004	
Lobe	−8.14	−14.87, −1.00	0.018	74	0.010	0.808
Hemostasis time	**−3**.**79**	**−5.93, −1.65**	**<0**.**001**	**91**	**<0**.**001**	
En-bloc	−2.58	−8.01, 2.85	0.352	48	<0.001	
Lobe	−4.08	−4.91, −3.25	<0.001	98	0.126	0.592
Operation time	**−12**.**09**	**−18.01, −6.18**	**<0**.**001**	**71**	**0**.**007**	
En-bloc	−10.41	−17.03, −3.78	0.002	79	0.009	
Lobe	−17.32	−23.02, −2.62	0.021	61	0.108	0.401
Enucleated weight	**1**.**01**	**−3.20, 5.21**	**0**.**639**	**72**	**<0**.**001**	
En-bloc	4.79	1.63, 7.96	0.003	50	0.111	
Lobe	−1.06	−8.79, 6.67	0.788	6	0.363	0.003
Length of Stay	**−0**.**23**	**−0.52, 0.05**	**0**.**113**	**85**	**<0**.**001**	
En-bloc	−0.11	−0.35, 0.12	0.348	87	<0.001	
Lobe	−0.58	−1.39, 0.23	0.159	82	0.019	0.275
Catheterization time	**−0**.**13**	**−0.35, 0.09**	**0**.**240**	**66**	**0**.**020**	
En-bloc	−0.10	−0.30, 0.11	0.340	68	0.040	
Lobe	−0.23	−1.01, 0.54	0.560	81	0.020	0.749
Complications CD 1	**0**.**44**	**0.30, 0.66**	**<0**.**001**	**7**	**0**.**368**	
En-bloc	0.39	0.24, 0.64	<0.001	51	0.154	
Lobe	0.53	0.23, 1.24	0.310	1	0.365	0.507
Complications CD 2	**0**.**58**	**0.45, 0.74**	**<0**.**001**	**14**	**0**.**322**	
En-bloc	0.54	0.41, 0.70	<0.001	17	0.271	
Lobe	1.00	0.38, 2.62	–		–	0.310
Complications CD ≥3	**0**.**40**	**0.16, 0.99**	**0**.**048**	**0**	**0**.**676**	
En-bloc	0.12	0.01, 0.99	0.049	0	0.536	
Lobe	0.76	0.25, 2.30	0.627	0	0.754	0.318

MD, mean difference; OR, odds ratio; CI, confidence interval; CD, Clavien–Dindo.

* *P* value for the test for subgroup difference.

**Table 3:** Results of the primary analysis and subgroup analyses by study design.

Outcomes	MD	95% CI	P	Heterogeneity		P* difference
				I² (%)	P	
Enucleation time	**−7**.**45**	**−11.05, −3.85**	**<0**.**001**	**75**	**<0**.**001**	**0**.**519**
RCT	−5.61	−9.10, −2.12	0.002	46	0.175	
Nonrandomized	−7.57	−12.40, −2.75	0.002	80	<0.001	
Hemostasis time	**−3**.**79**	**−5.93, −1.65**	**<0**.**001**	**91**	**<0**.**001**	**0**.**216**
RCT	−6.51	−11.57, −1.45	0.012	63	0.099	
Nonrandomized	−2.97	−5.39, −0.55	0.016	94	<0.001	
Operation time	**−12**.**09**	**−18.01, −6.18**	**<0**.**001**	**71**	**0**.**007**	**0**.**047**
RCT	−25.00	−38.31, −11.69	<0.001		–	
Nonrandomized	−10.47	−15.81, −5.13	<0.001	68	0.025	
Enucleated weight	**1**.**01**	**−3.20, 5.21**	**0**.**639**	**72**	**<0**.**001**	**0**.**590**
RCT	−2.54	−16.55, 11.47	0.722	65	0.090	
Nonrandomized	1.53	−3.15, 6.20	0.522	75	<0.001	
Catheterization time	**−0**.**13**	**−0.35, 0.09**	**0**.**238**	**66**	**0**.**019**	**0**.**748**
RCT	−0.23	−1.01, 0.54	0.559	81	0.022	
Nonrandomized	−0.10	−0.30, 0.11	0.340	68	0.043	
Length of stay	**−0**.**23**	**−0.52, 0.05**	**0**.**113**	**85**	**<0**.**001**	**0**.**275**
RCT	−0.58	−1.39, 0.23	0.159	82	0.018	
Nonrandomized	−0.11	−0.35, 0.12	0.347	87	<0.001	

MD, mean difference; CI, confidence interval; RCT, randomized controlled trial.

* *P* value for the test for subgroup difference.

### 3.4 Complications

Regarding early postoperative complications, pooled data from six studies (967 MoLEP vs. 1,009 HoLEP) showed a significantly lower incidence of Clavien–Dindo grade I complications with MoLEP (OR 0.44, 95% CI 0.30–0.66, *p* < 0.001, I^2^ = 7.5%; Figure 4A). Similarly, pooled analysis of four studies (885 MoLEP vs. 897 HoLEP) demonstrated a lower incidence of Clavien–Dindo grade II complications with MoLEP (OR 0.58, 95% CI 0.45–0.74, *p* < 0.001, I^2^ = 14.1%; Figure 4B). For Clavien–Dindo grade ≥ III complications, five studies (939 MoLEP vs. 939 HoLEP) showed a lower pooled risk with MoLEP (OR 0.40, 95% CI 0.16–0.99, *p* = 0.048, I^2^ = 0%; Figure 4C), although the confidence interval approached the null and the absolute number of major complications was limited.

**Figure 4:**
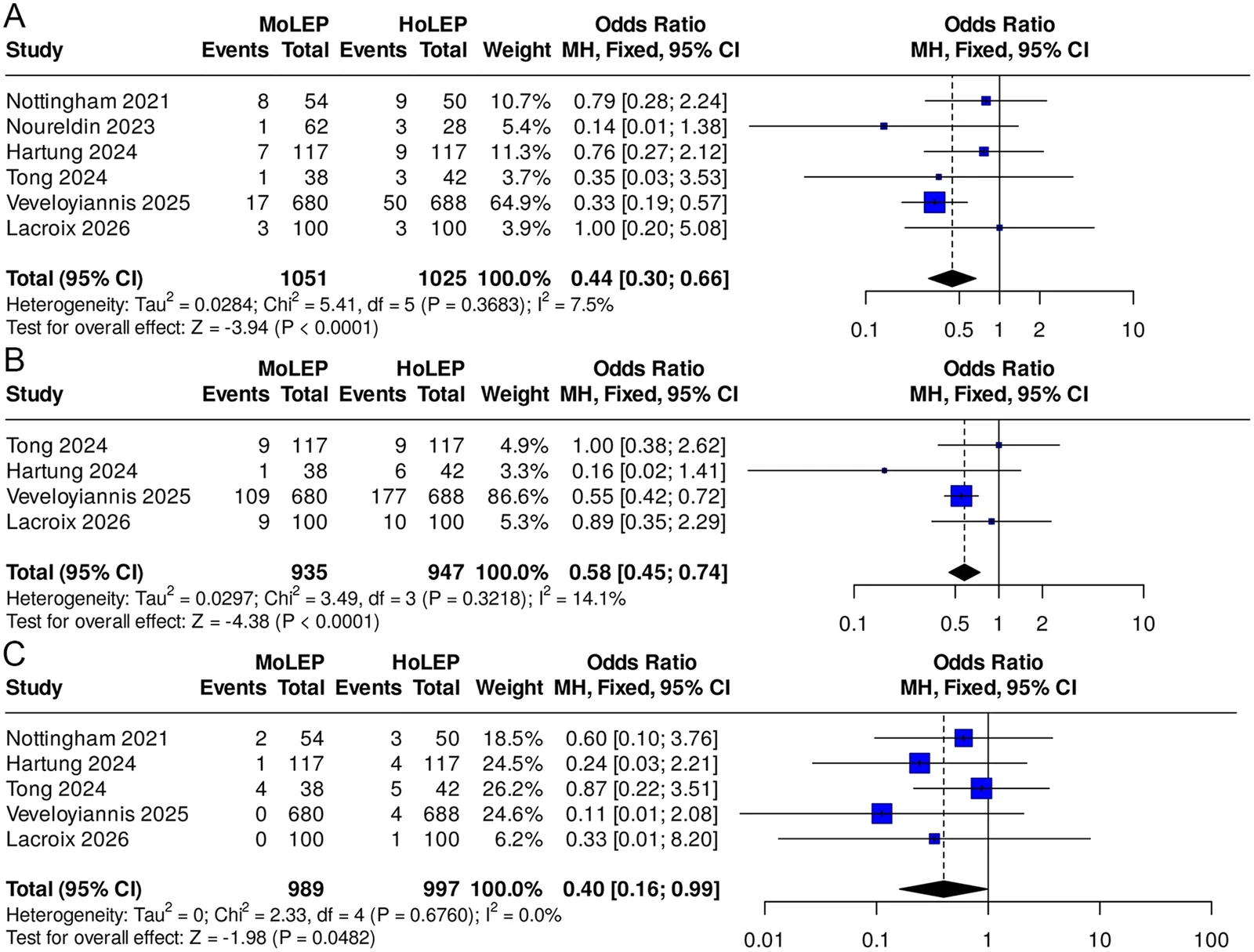
Forest plot representing analysis of complications. **(A)** complications Clavien-Dindo I, **(B)** complications Clavien-Dindo II, **(C)** complications Clavien-Dindo ≥ III.

In technique-based subgroup analyses, the direction of effect generally favored MoLEP in both en-bloc and lobe-based approaches. For grade I complications, the pooled ORs were 0.39 (95% CI 0.24–0.64) in the en-bloc subgroup and 0.53 (95% CI 0.23–1.24) in the lobe-based subgroup. For grade II complications, the corresponding ORs were 0.54 (95% CI 0.41–0.70) and 1.00 (95% CI 0.38–2.62), respectively. For grade ≥ III complications, the pooled ORs were 0.12 (95% CI 0.01–0.99) in the en-bloc subgroup and 0.76 (95% CI 0.25–2.30) in the lobe-based subgroup. However, tests for subgroup differences were not significant for grade I (*P* for interaction = 0.507), grade II (*P* for interaction = 0.310), or grade ≥ III complications (*P* for interaction = 0.318), providing no evidence that enucleation technique significantly modified the comparative effect of MoLEP on postoperative complications (Table 2).

### 3.5 Functional outcomes

During the 1-month follow-up (Figure 5), no significant between-group differences were observed in IPSS (MD −1.34, 95% CI −3.29–0.60, *P* = 0.18, I^2^ = 97%), QoL (MD −0.31, 95% CI −0.63 to −0.01, *P* = 0.06, I^2^ = 93.1%), or Qmax (MD 2.04, 95% CI −1.39–5.47, *P* = 0.24, I^2^ = 98.7%), whereas PVR was significantly lower with MoLEP (MD −1.29 mL, 95% CI −1.99 to −0.58, *P* < 0.001, I^2^ = 41.5%). During the 3-month follow-up (Figure 6), IPSS did not differ significantly between groups (MD −0.89, 95% CI −2.46–0.68, *P* = 0.27, I^2^ = 91.9%), whereas the QoL score was significantly lower with MoLEP (MD −0.17, 95% CI −0.23 to −0.11, *P* < 0.001, I^2^ = 0%). No significant between-group differences were observed in Qmax (MD −0.89, 95% CI −4.28–2.51, *P* = 0.61, I^2^ = 87.0%) or PVR (MD 4.13 mL, 95% CI −5.19–13.44, *P* = 0.39, I^2^ = 70.9%). During the 6-month follow-up (Figure 7), IPSS was significantly lower with MoLEP (MD −0.77, 95% CI −1.47 to −0.07, *P* = 0.03, I^2^ = 74.4%), whereas QoL (MD −0.04, 95% CI −0.12–0.05, *P* = 0.40, I^2^ = 0%) and Qmax (MD 0.67, 95% CI −3.86–5.20, *P* = 0.77, I^2^ = 99.6%) did not differ significantly between groups. PVR was also modestly lower with MoLEP (MD −2.57 mL, 95% CI −5.08 to −0.05, *P* = 0.045, I^2^ = 94.6%). Taken together, statistically significant between-group differences were observed for PVR at 1 and 6 months, QoL at 3 months, and IPSS at 6 months; however, the absolute magnitudes of these differences were small. The 6-month difference in IPSS (MD −0.77) was well below the commonly cited 3-point threshold for a clinically meaningful change (28), while the 3-month difference in QoL (MD −0.17) was also substantially smaller than the commonly used 0.5-point benchmark (29). Although PVR was statistically lower with MoLEP at 1 month (MD −1.29 mL) and 6 months (MD −2.57 mL), these absolute differences were small, and the 6-month estimate was accompanied by substantial heterogeneity (I^2^ = 94.6%). No consistent between-group advantage was observed for Qmax. Overall, these findings suggest that the observed differences in early functional outcomes are modest and unlikely to represent substantial clinically meaningful benefits.

**Figure 5:**
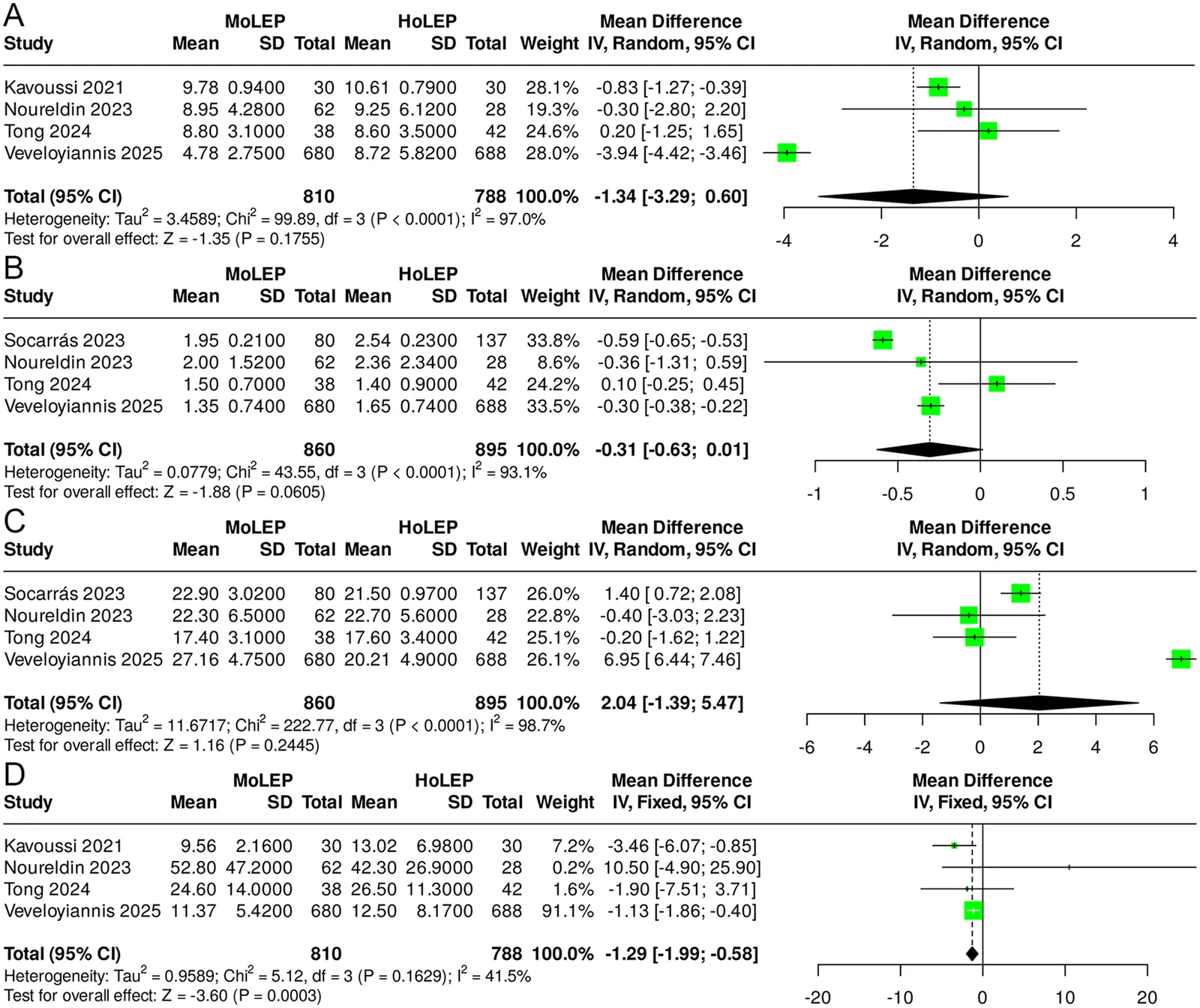
Forest plot representing analysis of 1-month postoperative functional outcomes. **(A)** International Prostate Symptom Score, **(B)** quality of life, **(C)** maximum urinary flow rate, and **(D)** postvoid residual volume.

**Figure 6:**
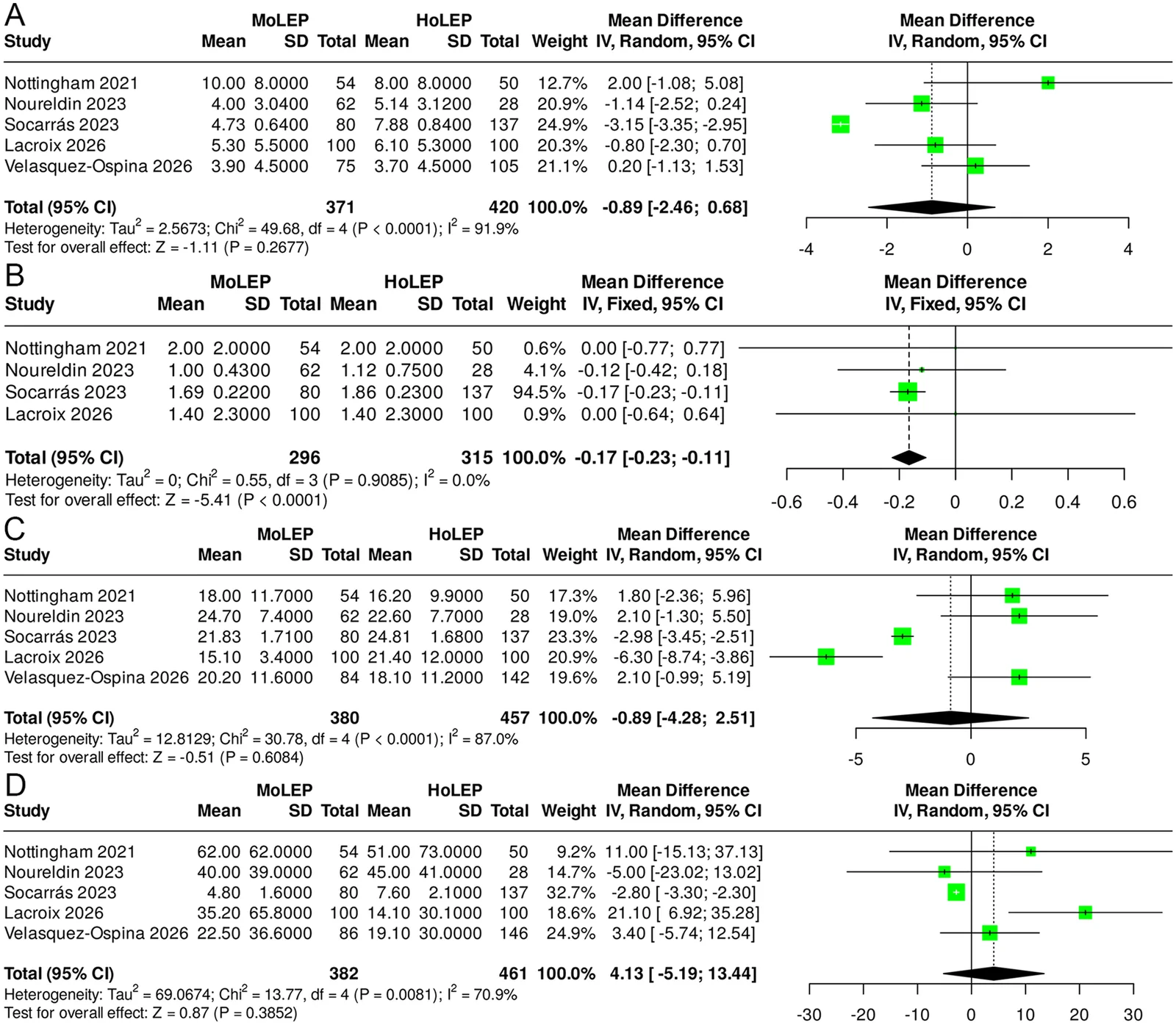
Forest plot representing analysis of 3-month postoperative functional outcomes. **(A)** International Prostate Symptom Score, **(B)** quality of life, **(C)** maximum urinary flow rate, and **(D)** postvoid residual volume.

**Figure 7:**
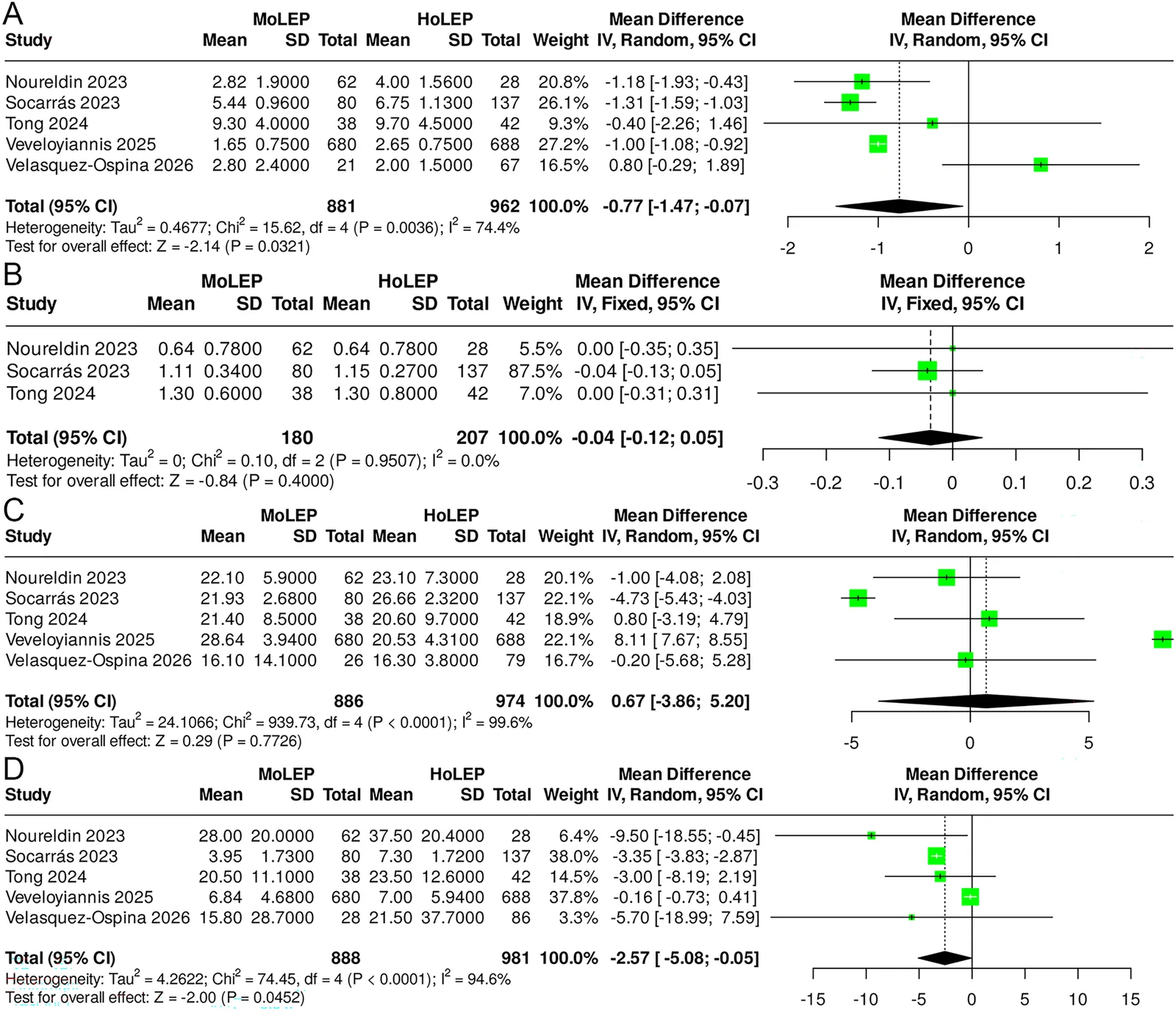
Forest plot representing analysis of 6-month postoperative functional outcomes. **(A)** International Prostate Symptom Score, **(B)** quality of life, **(C)** maximum urinary flow rate, and **(D)** postvoid residual volume.

### 3.6 Sensitivity analysis and publication bias

Leave-one-out sensitivity analyses demonstrated that the pooled findings for enucleation time, hemostasis time, and operative time were robust, with the direction and statistical significance of the effects remaining unchanged after sequential exclusion of individual studies. Similarly, the nonsignificant findings for enucleated weight and catheterization time were unchanged in all leave-one-out analyses. The pooled estimate for length of stay was less robust: although the primary analysis showed no significant difference between groups, exclusion of Velasquez-Ospina et al. resulted in a statistically significant reduction with MoLEP (SMD −0.31, 95% CI −0.62 to −0.01, *p* = 0.043). Because fewer than 10 studies were available for each outcome, assessment of publication bias was limited. Exploratory funnel plots did not show a consistent pattern of marked asymmetry across the principal perioperative outcomes. Exploratory Egger regression was nonsignificant for operative time, enucleation time, hemostasis time, catheterization time, and length of stay (all *p* > 0.05), whereas possible small-study asymmetry was observed for enucleated weight (*p* = 0.034). These findings should be interpreted cautiously because of the small number of studies.

## 4 Discussion

In this updated meta-analysis, MoLEP was associated with greater intraoperative efficiency than conventional HoLEP, particularly through shorter enucleation and hemostasis times, together with a modest reduction in overall operative time. MoLEP was also associated with a generally lower incidence of postoperative complications across Clavien–Dindo categories. In contrast, postoperative functional outcomes were largely similar. These findings suggest that the principal benefit of MOSES pulse modulation lies in incremental improvements in procedural efficiency and minor postoperative morbidity rather than in substantial short-term functional gains.

The perioperative advantages observed with MoLEP are biologically plausible in terms of laser–fluid–tissue interaction. MOSES technology modulates Ho:YAG pulse delivery into sequential sub-pulses; the initial component creates a vapor channel, allowing subsequent energy to propagate more efficiently through the fluid medium and potentially improving energy coupling at the target surface (5, 30). Although this physical mechanism was initially characterized mainly in lithotripsy models, the same pulse-modulation principle may facilitate more controlled tissue separation during HoLEP. Consistent with this interpretation, Nevo et al. (31) reported less fibre degradation and higher subjective ratings for incision sharpness, fibre control, tissue separation, haemostasis, visibility, and charring with MOSES 2.0, together with shorter enucleation time in expert-performed procedures and shorter haemostasis laser time in trainee-performed procedures. Kavoussi et al. (8) similarly demonstrated shorter operative, enucleation, and haemostasis times and a smaller postoperative hematocrit reduction with MOSES-assisted HoLEP. Importantly, these haemostatic advantages should not be interpreted as evidence of deeper thermal injury. In *ex vivo* human prostate tissue, coagulation-necrosis depth was similar between MOSES and conventional long-pulse holmium laser over a broad range of power settings (32). Thus, the observed efficiency gains may primarily reflect altered energy delivery, improved fibre stability and visualization, and more controlled tissue handling rather than greater tissue penetration or tissue enucleation.

Three previous meta-analyses have evaluated MoLEP versus conventional HoLEP. Gauhar et al. (11) and Ramadhani et al. (13) each included seven studies and reported advantages of MOSES technology in enucleation and hemostasis efficiency, while postoperative functional outcomes were generally comparable; Ramadhani et al. (13) additionally reported a lower pooled PVR with MoLEP. Li et al. (12) subsequently included 12 reports involving 1,160 patients from both international and Chinese databases and similarly identified perioperative advantages, although the pooled QoL result differed in direction from our time-specific analysis. The present study extends these earlier analyses by incorporating more recent comparative evidence and reassessing the independence of the underlying cohorts. Cross-checking institutional sources and recruitment periods identified potentially overlapping Indiana University cohorts, and only one representative cohort was retained to minimize duplicate patient representation. Newly published comparative cohorts, including Lacroix et al. (26) and Velasquez-Ospina et al. (27) in 2026, were also incorporated. Together with follow-up-specific analyses of functional outcomes, these updates provide a more current and methodologically rigorous synthesis of the comparative evidence.

Subgroup analyses provided limited evidence that study design or enucleation technique consistently modified the comparative effects of MoLEP. The reductions in enucleation and hemostasis times were generally consistent across both en-bloc and lobe-based approaches and across RCTs and nonrandomized studies, with no significant subgroup interactions. A significant technique-based interaction was observed for enucleated weight, with a greater enucleated weight in the en-bloc subgroup but no significant difference in the lobe-based subgroup. Operative time also differed according to study design, with a larger reduction in the RCT subgroup; however, this finding should be interpreted cautiously because the RCT subgroup contained only one study. Overall, these findings suggest that neither enucleation technique nor study design alone consistently explains the observed between-study heterogeneity.

Early functional outcomes should be interpreted cautiously. Although several between-group differences reached statistical significance at individual follow-up time points, these effects were small, inconsistent over time, and did not show a uniform pattern across symptom scores, urinary flow, and residual urine. In particular, the reduction in IPSS at 6 months remained well below the commonly cited threshold for a clinically meaningful change (28), while the observed difference in QoL was also substantially smaller than the commonly used 0.5-point benchmark (29). This is consistent with recent BPH literature emphasizing that statistically significant differences in postoperative symptom scores do not necessarily indicate clinically meaningful superiorit (33). Similarly, the modest reductions in PVR observed at selected time points are unlikely to be clinically important, particularly given the substantial heterogeneity at later follow-up. The absence of a consistent effect on Qmax further suggests that MOSES pulse modulation does not materially alter early postoperative functional recovery. Taken together, the functional benefits of MoLEP appear limited in magnitude and temporal consistency, supporting an interpretation of broadly comparable early functional outcomes between MoLEP and conventional HoLEP.

Although several perioperative endpoints reached statistical significance, the absolute differences were modest and are unlikely to produce substantial perceptible benefit for an individual patient. Their relevance may instead be greatest at the system level, particularly in high-volume centers, where small reductions in procedure or hemostasis time may accumulate across multiple cases. Similarly, fewer low-grade complications may reduce minor postoperative interventions or unscheduled contacts even when major complication rates remain unchanged. Thus, the clinical value of MOSES technology appears to lie primarily in incremental procedural efficiency and minor morbidity rather than in marked improvements in short-term symptom recovery.

The substantial heterogeneity observed for several outcomes likely reflects differences in prostate size, surgeon experience and learning-curve stage, enucleation technique, laser platform and pulse settings, fibre characteristics, perioperative pathways, and definitions of efficiency outcomes. Operator experience and prostate size are particularly important determinants of operative efficiency and may modify the apparent benefit attributable to pulse modulation (34–38). Because subgroup analyses did not identify a consistent modifying effect of either study design or enucleation technique across outcomes, the observed heterogeneity is likely multifactorial. Standardized definitions of enucleation, hemostasis, and operative time will therefore be important for future comparative studies. Leave-one-out sensitivity analyses supported the robustness of the principal efficiency findings, as the reductions in enucleation, hemostasis, and operative times remained significant after sequential exclusion of individual studies. In contrast, the pooled estimate for length of stay was sensitive to exclusion of one recent cohort and should therefore be interpreted more cautiously. Assessment of small-study effects was limited by the small number of studies available for each outcome; exploratory analyses did not suggest a consistent pattern of asymmetry, although possible asymmetry was observed for enucleated weight.

Several limitations should be acknowledged. First, one RCT was judged to be at low risk of bias and the other as having some concerns under RoB 2, while the nonrandomized comparative studies ranged from moderate to serious risk of bias under ROBINS-I. These limitations, including residual confounding, nonrandomized treatment allocation, historical control designs, and missing follow-up data in some studies, reduce confidence in the pooled estimates. This is particularly relevant for subjective outcomes such as IPSS and QoL, which may also be susceptible to expectation effects when treatment allocation is not blinded. Second, substantial heterogeneity was present for several outcomes, reflecting differences in patient characteristics, operative technique, surgeon experience, laser settings, and outcome definitions. Third, economic outcomes could not be meaningfully synthesized in the revised evidence base, and formal cost-effectiveness analyses remain lacking. Fourth, follow-up was predominantly short-term, with limited evidence beyond 6–12 months, restricting conclusions regarding durability, late complications, retreatment, and long-term patient-reported outcomes. Future randomized or well-matched prospective studies with standardized outcome definitions, transparent reporting of laser and surgical parameters, and longer follow-up are warranted.

## 5 Conclusion

Overall, MOSES pulse modulation appears to provide incremental improvements in intraoperative efficiency and is associated with a generally lower incidence of postoperative complications, while early functional outcomes remain broadly comparable with conventional HoLEP. These findings support MOSES-assisted HoLEP as a reasonable contemporary treatment option, although higher-level evidence is still needed to confirm the magnitude, durability, and clinical relevance of these potential benefits.

## Data Availability

The original contributions presented in the study are included in the article/Supplementary Material, further inquiries can be directed to the corresponding authors.
