# Supplementary material for: MOSES-assisted versus conventional holmium laser enucleation of the prostate for benign prostatic hyperplasia: a pooled study
Source: Front Med (Lausanne). 2026 Oct 14;13:1946428. doi: 10.3389/fmed.2026.1946428 (PMC13713476; doi:10.3389/fmed.2026.1946428)
Supplement: Supplementary file 1 [file Datasheet1.docx]

**Figure S1.** The risk of bias assessment of included studies. (A) randomized controlled trial, (B)Non- randomized controlled trial.
